# MiR-150-5p suppresses high-grade transformation of lacrimal adenoid cystic carcinoma by downregulating COL1A1

**DOI:** 10.3389/fonc.2026.1915447

**Published:** 2026-08-26

**Authors:** Xiaoyu Zhu, Jinjie Liu, Meixia Jiang, Xun Liu, Shuang Chen, Tingting Lin, Limin Zhu

**Affiliations:** 1 Tianjin Key Laboratory of Retinal Functions and Diseases, Tianjin Branch of National Clinical Research Center for Ocular Disease, Eye Institute and School of Optometry, Tianjin Medical University Eye Hospital, Tianjin, China; 2 Beijing Tongren Eye Center, Beijing Tongren Hospital, Capital Medical University, Beijing Key Laboratory of Ophthalmology & Visual Sciences, Beijing, China; 3 Tianjin Key Laboratory of Early Drug Evaluation of Innovative Drugs, Tianjin International Joint Academy of Biomedicine, Tianjin, China

**Keywords:** COL1A1, epithelial-mesenchymal transition, high-grade transformation, lacrimal gland adenoid cystic carcinoma (LACC), miR-150-5p

## Abstract

Lacrimal adenoid cystic carcinoma (LACC) is the most common malignant epithelial tumor of the lacrimal gland, with particularly high recurrence and mortality in cases with high-grade transformation (HGT-LACC). This study aims to investigate the functional role of miR-150-5p in regulating the HGT of LACC. MiRNA microarray and proteomic mass spectrometry were performed on paired LACC and HGT-LACC tissues to identify differentially expressed miR-150-5p and its potential target COL1A1. The miR-150-5p/COL1A1 interaction was validated by luciferase reporter assay, and the effects of miR-150-5p on proliferation, migration, invasion, and epithelial-mesenchymal transition (EMT) were assessed *in vitro* and *in vivo* via colony formation, wound healing, Transwell assays, and xenograft models. MiR-150-5p was significantly downregulated in HGT-LACC and correlated with poor prognosis. Overexpression of miR-150-5p suppressed cell proliferation, migration and invasion, while its knockdown showed opposite effects. COL1A1 was confirmed as the direct target of miR-150-5p, and COL1A1 restoration counteracted the tumor-suppressive effect of miR-150-5p. *In vivo*, miR-150-5p overexpression inhibited tumor growth. Overall, our findings demonstrate that miR-150-5p suppresses LACC cell proliferation, migration, invasion, and EMT by downregulating COL1A1 expression, thereby functioning as a tumor suppressor. The miR-150-5p-COL1A1 axis provides novel insights into the pathogenesis of LACC and serves as a promising therapeutic target for LACC intervention.

## 1 Introduction

Studies have demonstrated that adenoid cystic carcinomas (ACCs) arising from different primary sites share similar gene expression profiles (1)and molecular mechanisms (2), which has been reported in the salivary gland (3), breasts (4), tracheobronchial tree (5), and Bartholin’s glands (6). LACC is recognized as the most frequent malignant epithelial neoplasm that originates from the lacrimal gland (7). In lacrimal gland tumor the incidence rate of LACC is lower than that of pleomorphic adenoma, accounting for approximately 25%–40% (8, 9). HGT is closely associated with tumor dedifferentiation and a marked increase in malignancy, ultimately resulting in poor prognosis for patients (10–13). However, the molecular mechanisms driving the malignant progression of LACC remain incompletely understood.

MiRNAs are a class of important endogenous small non-coding RNAs that play key roles in various biological processes such as cell proliferation, apoptosis, differentiation, and invasion by negatively regulating the expression of target genes at the post-transcriptional level (14, 15). A growing body of evidence indicates that the aberrant expression of miRNAs is a key factor in the tumorigenesis and progression of numerous malignant tumors (16). However, research on the role of miRNAs in HGT-LACC remains extremely scarce to date.

Previously, our research group performed miRNA microarray analysis on clinical tissue samples of LACC and HGT-LACC, and miR-150-5p was identified as one of the key molecules significantly downregulated in HGT-LACC. To further identify its downstream functional targets, we conducted proteomic analysis on the same clinical samples, which revealed that COL1A1 was significantly upregulated in HGT-LACC. The central focus of this study was to delineate the critical molecular mediators that drive malignant transformation events in HGT-LACC, with special attention devoted to examining the functional role of miR-150-5p and its potential molecular interaction with *COL1A1*.

## 2 Materials and methods

### 2.1 Ethical considerations and informed consent

All biological samples collected for the present study were strictly restricted to use in relevant scientific research projects. Formal written informed consent was duly obtained from every participating patient prior to sample collection. This study has been approved by the Human Research Ethics Committee of Tianjin Medical University Eye Hospital (Tianjin, China; approval no. 2017KY(L)-23) and complied with the Declaration of Helsinki.

### 2.2 Sample collection

Paraffin samples were divided into LACC and HGT-LACC groups via pathological diagnosis. 12 paraffin samples (6 LACC and 6 HGT-LACC) were collected for miRNA gene chip analysis. 8 samples (4 LACC and 4 HGT-LACC) were selected for iTRAQ-based proteomics analysis combined with liquid chromatography–tandem mass spectrometry (LC-MS/MS). In addition, 39 LACC patients were enrolled, consisting of 31 non-HGT cases and 8 HGT-LACC cases. Paraffin-embedded tissues were collected, and comprehensive clinicopathological parameters alongside long-term follow-up prognostic data were retrospectively collated.

### 2.3 MiRNA gene chip analysis

6 LACC and 6 HGT-LACC paraffin-embedded tissue samples were used in the miRNA gene chip analysis. Total RNAs were extracted from paraffin tissues using the RecoverAllTM Total Nucleic Acid Isolation Kit. The purified RNA samples underwent integrity quality assessment utilizing the Agilent Bioanalyzer 2100 platform (Agilent Technologies). Total RNAs were dephosphorylated, desaturated and then labeled with Cyanine-3-CTP. After purification the labeled RNAs were hybridized onto the microarray. Differentially expressed miRNAs were detected in the extracted RNAs by Agilent human miRNA microarray (Release 21.0, 8*60K, Design ID:070156). After washing, the arrays were scanned with the Agilent Scanner G2505C (Agilent Technologies). All protocols were performed based on the manufacturer’s standard protocols.

### 2.4 Mass spectrometry

4 LACC and 4 HGT-LACC paraffin-embedded tissue samples was performed to compare the differential proteins qualitatively and quantitatively. After paraffin tissue was deparaffinized, protein extraction and repair were performed. Protein samples were enzymatically digested into peptides, and different iTRAQ tags were used to label different peptide samples. Protein analysis was performed using a Thermo Q-Exactive type mass spectrometer, and LC-MS/MS was performed for each component. Raw files of mass spectra were processed using ProteinPilot software and protein searches were performed using UniProt, Homo sapiens databases. The protein expression data were statistically analyzed. Proteins exhibiting a P value less than 0.05 and a fold change (FC) greater than 1.5 were identified as differentially expressed proteins according to the predefined selection criteria.

### 2.5 Cell culture

To date, only mature and usable cell lines have been established for salivary adenoid cystic carcinoma (SACC). Two SACC cell lines, SACC-83 and SACC-LM, were used in this study for *in vitro* and *in vivo* experiments. Both cell lines were derived from a 26-year-old female patient in 1983, but SACC-LM is a subclone with high metastatic potential. They were purchased from ZQXZbio (Shanghai, China) and cultured in RPMI-1640 medium supplemented with 10% fetal bovine serum (FBS) and 1% penicillin-streptomycin, in a humidified incubator at 37 °C with 5% CO_2_.

### 2.6 Quantitative reverse transcriptase polymerase chain reaction

Total miRNAs were isolated from tissues and cells using the Universal microRNA Purification Kit (EZBioscience, USA). Then, the miRNAs were reverse-transcribed into complementary DNA (cDNA) using the EZ-press microRNA Reverse Transcription Kit (EZBioscience, USA). For quantitative detection, the EZ-press microRNA qPCR Kit (ROX1) (EZBioscience, USA) along with its corresponding specific primers were utilized. 2^−ΔΔCT^ method was used to determine expression level, internal control is U6. The primers employed in these experiments are listed below: miR-150-5p forward primer: 5ʹ-CGCTCTCTCCCAACCCTTGTACC-3ʹ, with the reverse primer being the universal primer provided by the commercial qPCR kit; U6 forward primer: 5ʹ- CTCGCTTCGGCAGCACA-3ʹ, reverse primer: 5ʹ-AACGCTTCACGAATTTGCGT-3′.

### 2.7 Cell transfection and lentiviral -mediated transduction

Target cells were seeded into 6-well culture plates and maintained under standard incubation conditions until the cell monolayer achieved 60–80% confluency. Following the standardized protocols provided by the respective manufacturers, a panel of oligonucleotides—including miR-150-5p mimics, miR-150-5p inhibitor, *COL1A1*-targeting small interfering RNA (siRNA), and their corresponding negative control oligonucleotides, all procured from Gene Pharma Co., Ltd. (Shanghai, China)—were introduced into the pre-cultured cells via lipofection using Lipofectamine 3000 transfection reagent (Invitrogen, USA). At 6 hours post-transfection, the culture medium was replaced with fresh medium, and the cells were subsequently incubated overnight.

Lentiviral vectors used in this study were obtained from OBIO Technology (Shanghai, China). When the cell confluence reached 30%–50%, the cells were cultured in complete RPMI 1640 medium containing 5 µg/mL polybrene, supplemented with an appropriate dose of lentivirus overexpressing *COL1A1* and its negative control. After 12 hours of transfection, the culture medium was replaced with fresh complete medium.

### 2.8 Wound healing assay

When the density of the transfected SACC-83 and SACC-LM cell lines grew to 80%–90%, a wound was induced via 1000μl sterile pipette tips. Cell migration was measured at 0 hours and 24 hours after scratching, and the healing rate was calculated. Each experiment was performed three times independently.

### 2.9 Invasion experiment

After transfection of the SACC-83 cell line and SACC-LM cell line for 48 hours, a certain number of cells (1×10^2^) were seeded into Transwell inserts. They were then cultured in a 37 °C, 5% CO_2_ incubator for 24 hours. Then, the matrixgel and cells on the membrane were removed, and the cells were stained with crystal violet for 30 minutes at room temperature. Three randomly selected fields were photographed under a microscope, and the number of successfully invaded cells was calculated.

### 2.10 Colony formation assay

The transfected SACC-83 cells and SACC-LM cells (1×10^3^) were cultured in a 37 °C, 5% CO_2_ incubator for 2–3 weeks. The culture was stopped when visible colonies appeared in the six-well plates. After fixation with methanol, the colonies were stained with crystal violet. The average number of colonies from three replicate wells was used to calculate the clonogenicity.

### 2.11 Western blot

The total protein of the cells was harvested with radioimmunoprecipitation assay buffer (Beyotime) containing phenylmethylsulfonyl fluoride (Solarbio, China). Protein concentration was measured using a bicinchoninic acid protein assay kit (Solarbio, China). Equal loads of total protein (50 μg per sample) from each experimental group underwent separation through 6% or 7.5% sodium dodecyl sulfate–polyacrylamide gel electrophoresis, with the separated proteins subsequently transferred onto polyvinylidene fluoride membranes (Millipore, USA). Following the transfer process, the membranes were treated with a 5% (w/v) nonfat milk blocking buffer for 2 hours under room temperature conditions. Thereafter, the prepared membranes were incubated with the corresponding primary antibodies (COL1A1, 1:2000 dilution, Proteintech Cat^#^: 14695-1-AP; E-cadherin, 1:500 dilution, Affinity Biosciences Cat^#^: AF0131; N-cadherin, 1:3000 dilution, Proteintech Cat^#^:22018-1-AP; Vimentin, 1:1500 dilution, Affinity Biosciences, Cat^#^: AF7013) and β-actin antibodies (1:8000 dilution, Affinity Biosciences Cat^#^: AF7018) overnight at 4 °C. The signals were detected after incubation with the corresponding horseradish peroxidase-conjugated secondary antibodies (1:7000 dilution, Affinity Biosciences, Cat^#^: S0001) using Omni-ECL™ Femto Light Chemiluminescence Kit (Epizyme Biotech, China) and chemiluminescence gel imaging system. The bands were analyzed using ImageJ software (National Institutes of Health, USA), and the target protein level was normalized to β-actin.

### 2.12 Luciferase reporter assay

Luciferase reporter assay was performed to determine the targeted relationship between miR-150-5p and *COL1A1*. The 3′ UTR fragments of wild-type and mutant *COL1A1* that contain the miR-150-5p binding site were amplified and cloned into the luciferase reporter vector to construct luciferase plasmid. Then co-transfecting the luciferase plasmid with miR-150-5p mimics or negative control via Lipofectamine 3000 (Thermo Fisher Scientific). Luciferase activities were measured 48 h after transfection using the Dual-Luciferase Reporter Gene Assay Kit (Keygen Biotech, China).

### 2.13 Fluorescence *in situ* hybridization

Microtome was used to cut paraffin-embedded tissues into 4 μm. The unstained specimen was baked for 10 min at 70 °C. The slides were then immersed twice in xylene for 10 min. Afterward, the sections were deparaffinized and rehydrated through an ethanol series and air-dried. MiR-150-5p probes were used to detect miR-150-5p expression level. Hybridized slides were purified three times in 50% formamide/2×saline sodium citrate (SSC) for 5 min at 42–50 °C and then washed three times in 1×SSC for 5 min at 42–50 °C. Finally, the slides were washed in 2×SSC at room temperature and allowed to dry in a dark room. Exactly 10 µL of 4′,6-diaminido-2-phenylindole was applied to counterstain the slides, and fluorescence microscope (Nikon Eclipse 55i, Japan) was used to enumerate the specimens. The tissues of each patient were stained with hematoxylin and eosin and then examined by an expert pathologist to mark the LACC and HGT-LACC areas.

### 2.14 *In vivo* experiments

BALB/c nude mice (body weight 18–20 g), purchased from GemPharmatech Laboratory(Jiangsu, China), were maintained under specific pathogen-free (SPF) conditions. All animal experimental procedures were approved by the Animal Ethics Committee of Tianjin Medical University Eye Hospital. Single-cell suspensions comprising 5 × 10^6^ SACC-83 or SACC-LM cells in the log phase of growth were administered via subcutaneous injection into the axillary region of five-week-old mice, for the specific purpose of constructing a stable *in vivo* tumor growth model. The longest length (L) and shortest width (W) of the tumors were measured daily. The volume of the allograft tumors was calculated using the following formula: tumor volume = (L×W^2^)/2. When the tumor volume reached approximately 100 mm^3^, the mice were randomly divided into treatment and control groups. Mice were administered PBS, miR-150-5p NC, or miR-150-5p agomir via intratumoral injection for two weeks. Subsequently, the mice were euthanized by cervical dislocation. Part of the tumor tissue was used for qRT-PCR to detect the expression level of miR-150-5p.

### 2.15 Statistical analysis

All data analysis and figure creation were completed using GraphPad Prism 8.0 software. Intergroup differences were compared using the student t-test or one-way ANOVA followed by Dunnett’s multiple comparison test. Kaplan-Meier curves were drawn to describe survival data, and log-rank tests were used to analyze intergroup differences. All experiments were independently repeated at least three times, and differences were considered statistically significant when p < 0.05. For comparisons among different treatment groups, statistical significance was designated using the following notation: *P < 0.05, **P < 0.01, ***P < 0.001, and ****P < 0.0001.

## 3 Results

### 3.1 Clinicopathological characteristics of patients

A total of 39 patients with pathologically confirmed lacrimal adenoid cystic carcinoma (LACC) were enrolled in this study, and their clinicopathological characteristics are summarized in **Table 1**. There were 15 males (38.5%) and 24 females (61.5%), with an age range of 20–75 years and a mean age of 44.3 ± 13.6 years. Eight patients (20.5%) developed high-grade transformation (HGT-LACC), while 31 patients (79.5%) did not (non-HGT-LACC).

**Table 1:** Clinicopathological characteristics of 39 patients with lacrimal adenoid cystic carcinoma.

Clinicopathological parameters	Categories	Total (n=39)	HGT-LACC (n=8)	Non-HGT-LACC (n=31)
Gender	Male	15 (38.5%)	2 (25.0%)	13 (41.9%)
	Female	24 (61.5%)	6 (75.0%)	18 (58.1%)
Age (years)	Mean ± SD	44.3 ± 13.6	42.6 ± 9.6	44.7 ± 14.7
Histological grade	Grade I–II	29 (74.4%)	0 (0.0%)	29 (93.5%)
	Grade III	10 (25.6%)	8 (100.0%)	2 (6.5%)
T stage	T2~T3	16 (41.0%)	2 (25.0%)	14 (45.2%)
	T4	23 (59.0%)	6 (75.0%)	17 (54.8%)
Local recurrence	Yes	21 (53.8%)	8 (100.0%)	13 (41.9%)
	No	18 (46.2%)	0 (0.0%)	18 (58.1%)
Distant metastasis	Yes	11 (28.2%)	4 (50.0%)	7 (22.6%)
	No	28 (71.8%)	4 (50.0%)	24 (77.4%)
Overall survival (months)	Mean ± SD	43.0 ± 32.1	31.0 ± 13.1	51.0 ± 38.7

Follow-up data showed that all patients in the HGT-LACC group were of histological grade III, with a 100% local recurrence rate, significantly higher than 41.9% in the non-HGT-LACC group. The mean follow-up/survival time of the entire cohort was 43.0 ± 32.1 months. The mean overall survival (OS) of HGT-LACC patients was 31.0 months, markedly shorter than 51.0 months in the non-HGT-LACC group, indicating that high-grade transformation is a critical adverse prognostic factor for LACC. Subsequent analyses will be conducted based on this cohort to explore the correlation between the expression levels of miR-150-5p and COL1A1 and patient prognosis.

### 3.2 Downregulation of miR-150-5p in patients with HGT-LACC and closely associated with poor prognosis

**Figure 1:**
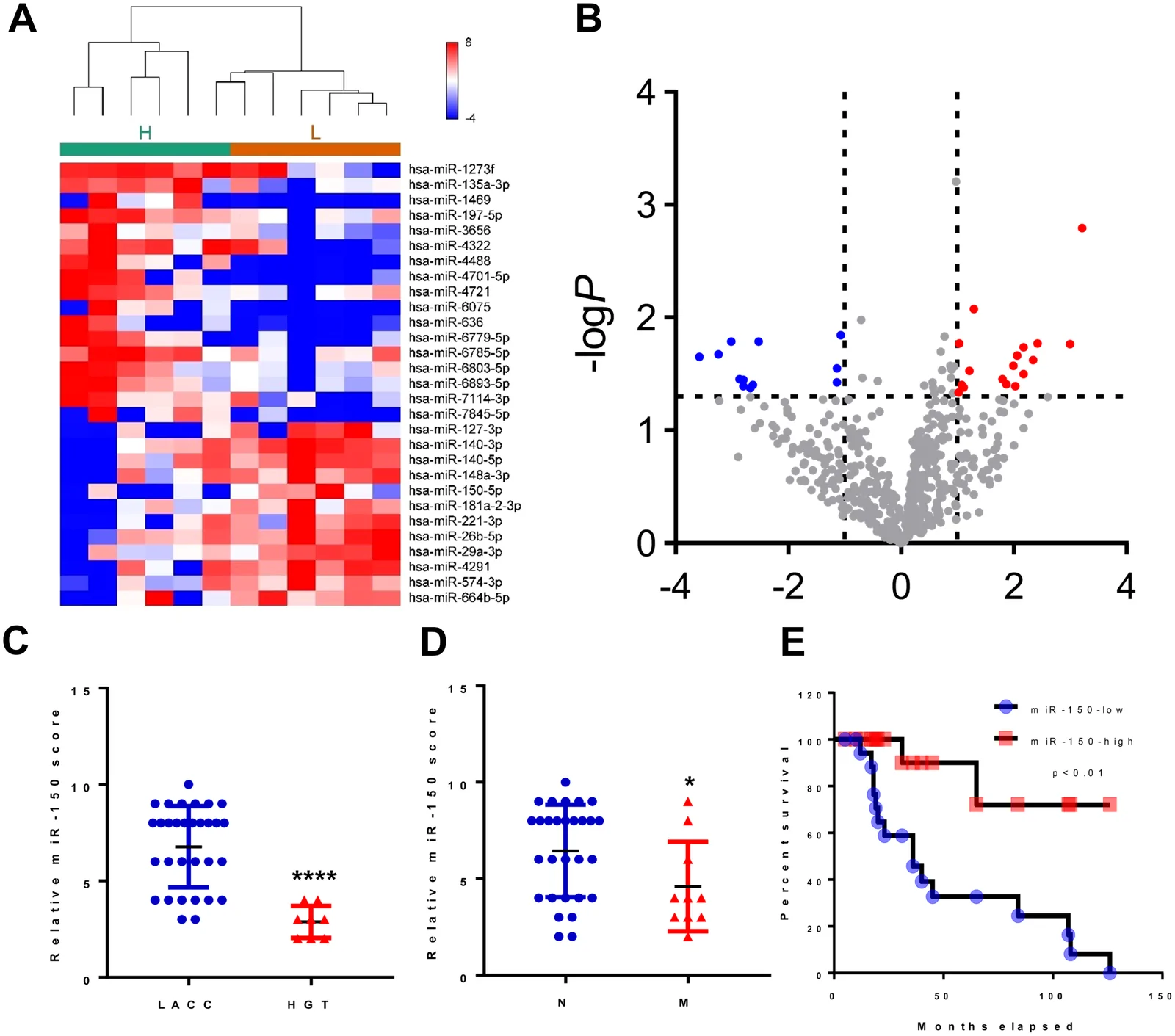
Downregulation of miR-150-5p in patients with HGT-LACC and closely associated with poor prognosis. **(A)** Heat map of the microRNA array of LACC and HGT-LACC. **(B)** Volcano graphs of differential miRNAs. **(C)** FISH staining results of miR-150-5p between LACC and HGT-LACC. **(D)** FISH staining results of miR-150-5p between LACC and metastasized LACC. **(E)** Survival rate of patients with different miR-150-5p expression levels. * indicates P < 0.05, and **** indicates P < 0.0001, representing statistically significant differences between the compared groups.

After the obtained data were standardized, we hierarchically clustered the differentially expressed miRNAs and visualized their expression patterns across different samples using heat maps (**Figure 1A**) and volcano plots (**Figure 1B**). A total of 24 differentially expressed miRNAs were identified in HGT-LACC paraffin-embedded tissues via miRNA microarray analysis. We found that the expression of miR-150-5p in HGT-LACC tumors was lower than that in LACC tumors. We performed FISH assay to detect miR-150-5p expression in 8 cases of HGT-LACC and 31 cases of LACC, as well as its relationship with disease prognosis. FISH staining results revealed that miR-150-5p expression in HGT-LACC was lower than that in LACC (**Figure 1C**), and its expression in metastatic LACC was lower than that in non-metastatic LACC (**Figure 1D**). Moreover, patients with low miR-150-5p expression exhibited poorer prognosis (**Figure 1E**).

### 3.3 Overexpression of COL1A1 in patients with HGT-LACC and potentially targeted by miR-150-5p

**Figure 2:**
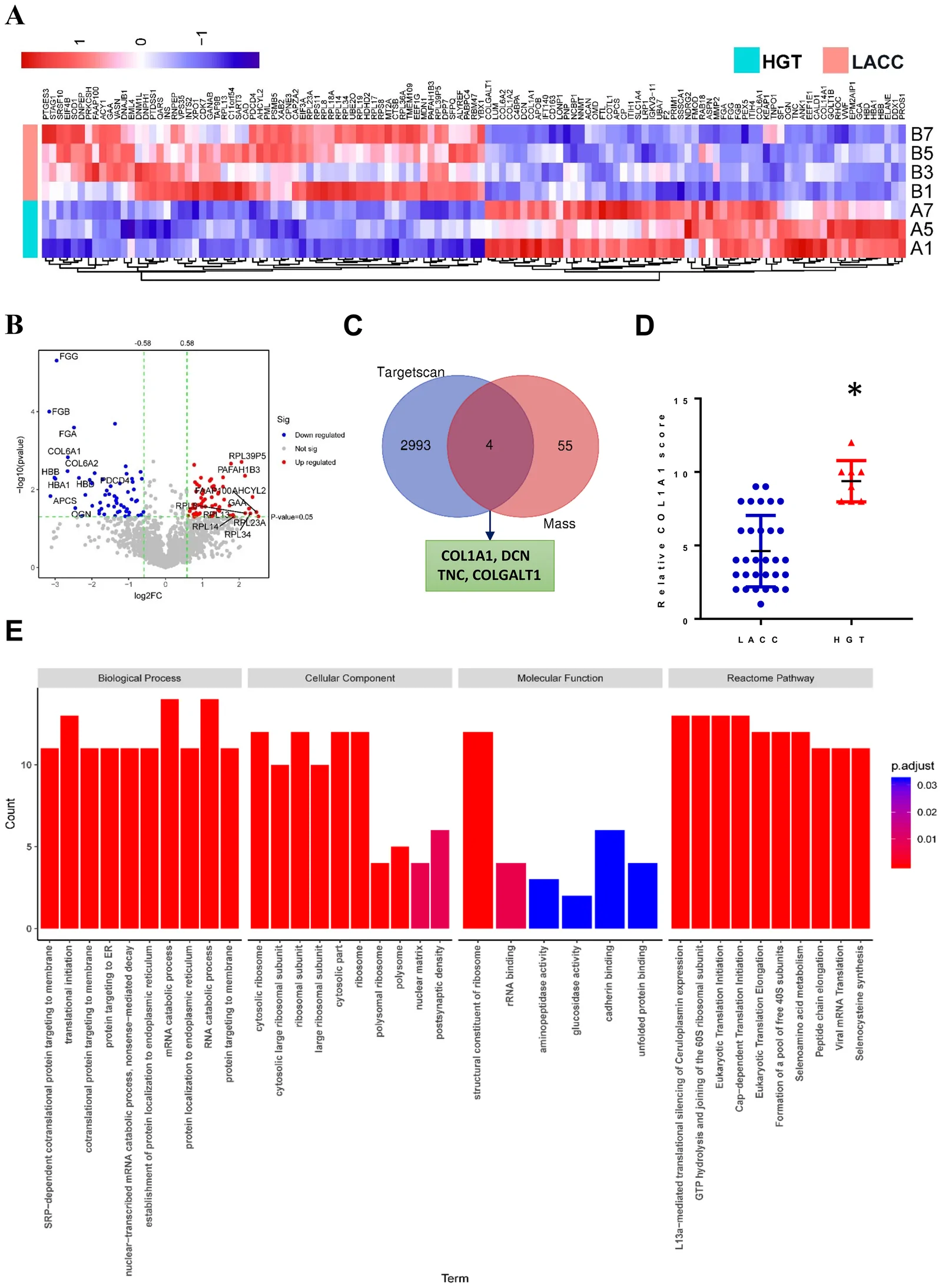
Overexpression of COL1A1 in patients with HGT-LACC and potentially targeted by miR-150-5p. **(A)** Heat maps of differential proteins in LACC and HGT-LACC. **(B)** Volcano graphs of differential proteins. **(C)** Venn diagram analysis of proteins upregulated in HGT-LACC according to the results of MS and target gene prediction by TargetScan. **(D)** COL1A1 expression in patients with LACC and HGT-LACC. **(E)** GO analysis of differential proteins identified via MS. * indicates P < 0.05, representing a statistically significant difference between the two groups.

The same eight pairs of LACC and HGT-LACC clinical samples were selected for proteomic analysis to explore the molecular mechanism of miR-150-5p in LACC progression. The expression of differential proteins is shown in the form of heat maps in **Figure 2A** and volcano maps in **Figure 2B**. We analyzed the upregulated proteins in HGT-LACC and the target gene of miR-150-5p predicted by the TargetScan database. Four genes, *COL1A1*, *DCN*, *TNC*, and *COLGALT1*, were identified from the proteomic data (**Figure 2C**). The results of immunohistochemical (IHC) analyses demonstrated that COL1A1 expression in HGT-LACC patients was markedly elevated in comparison with that detected in LACC patients (**Figure 2D**). These results were consistent with the MS and functional analysis of miR-150-5p. Additionally, Gene Ontology (GO) enrichment coupled with pathway analysis were conducted on the upregulated differential proteins in HGT-LACC (**Figure 2E**).

### 3.4 COL1A1 regulated by miR-150-5p and correlated with the poor prognosis

**Figure 3:**
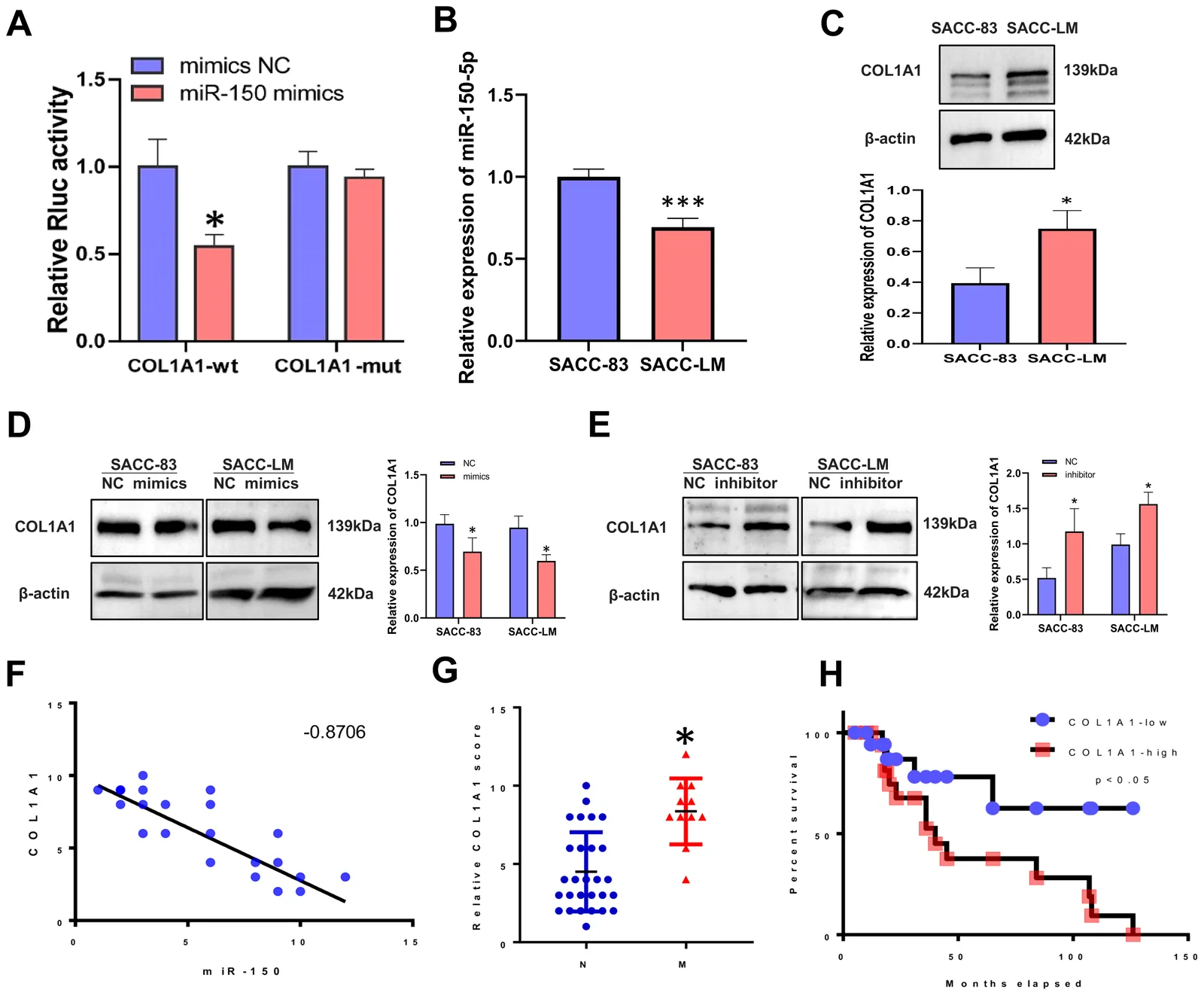
COL1A1 regulated by miR-150-5p and correlated with the poor prognosis. **(A)** Luciferase activity assay. **(B)** MiR-150-5p expression level in untreated SACC-83 and SACC-LM cells. **(C, D, E)** Western blot analysis of COL1A1 protein levels. **(F)** Correlation between miR-150-5p and COL1A1 in patients with LACC. **(G)** COL1A1 IHC staining results between LACC and metastasized LACC. **(H)** Survival rate of patients with different COL1A1 expression levels. * indicates P < 0.05, and *** indicates P < 0.001, representing statistically significant differences between the compared groups.

The results indicate that *COL1A1* may be a target gene of miR-150-5p. To validate this hypothesis, we constructed recombinant plasmids by inserting wild-type and mutant *COL1A1* 3’UTR sequences into a commercial luciferase reporter system. The obtained experimental data demonstrated that miR-150-5p could bind to *COL1A1* 3’UTR and inhibit luciferase expression (**Figure 3A**). Thereafter, we detected the expression differences of miR-150-5p and COL1A1 between untreated SACC-83 and SACC-LM cells. Experimental findings revealed that the expression level of miR-150-5p in SACC-LM was lower than that in SACC-83, while the expression level of COL1A1 was higher than that in SACC-83, consistent with our sequencing results(**Figures 3B, C**). Subsequently, we overexpressed or knocked down miR-150-5p in SACC-83 and SACC-LM cells and detected the expression of COL1A1 via Western blot to verify the targeting relationship between COL1A1 and miR-150-5p. Experimental results indicated that miR-150-5p could inhibit the expression of COL1A1 (**Figures 3D, E**). Additionally, we used IHC to detect the expression of COL1A1 in LACC and HGT-LACC tissues. The obtained experimental data demonstrated that the expression of COL1A1 was inversely correlated with that of miR-150-5p (**Figure 3F**) and was upregulated in HGT-LACC (and metastatic tumors) (**Figure 3G**). Furthermore, high expression of COL1A1 was closely related to poor prognosis in LACC patients (**Figure 3H**).

### 3.5 MiR-150-5p exerted tumor-suppressive effects

**Figure 4:**
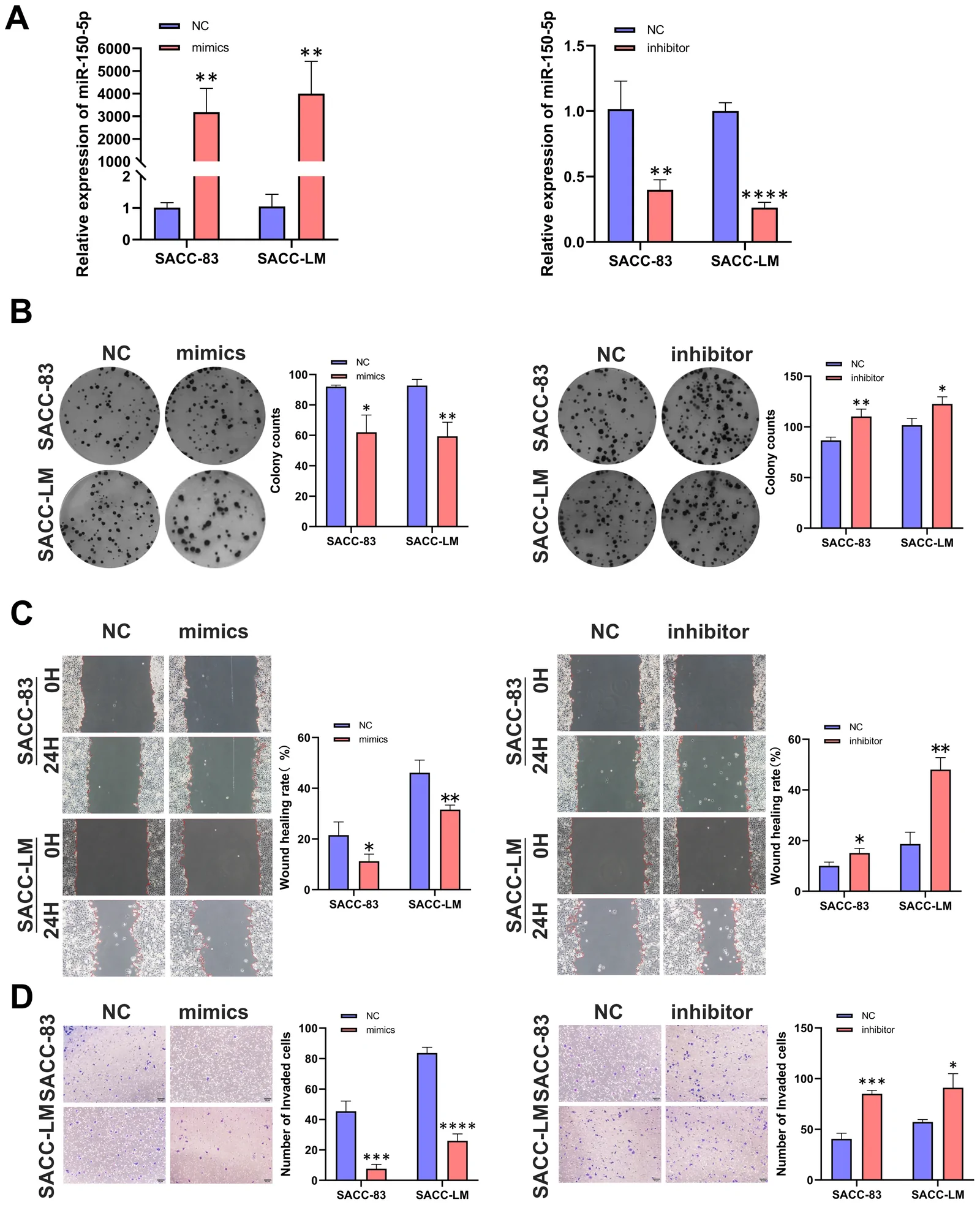
MiR-150-5p exerted tumor-suppressive effects. **(A)** MiR-150-5p expression level in SACC-83 and SACC-LM cells treated with miR-150-5p mimics or miR-150-5p inhibitor. **(B)** Colony formation assay, **(C)** Wound-healing assay, **(D)** Transwell invasion assay of SACC-83 and SACC-LM after miR-150-5p mimics or miR-150-5p inhibitor transfection. * indicates P < 0.05, ** indicates P < 0.01, *** indicates P < 0.001, and ns indicates no statistically significant difference between groups.

SACC-83 and SACC-LM cells with miR-150-5p overexpression or knockdown were established via transfection with miR-150-5p mimics or inhibitors, respectively (**Figure 4A**). Results from colony formation assay (**Figure 4B**), wound healing assay (**Figure 4C**) and Transwell invasion assay (**Figure 4D**) demonstrated that miR-150-5p overexpression suppressed the proliferation, migration and invasion abilities of SACC-83 and SACC-LM cells, whereas miR-150-5p knockdown promoted these cellular processes.

### 3.6 Overexpression of COL1A1 promoted the proliferation, migration, and invasion

**Figure 5:**
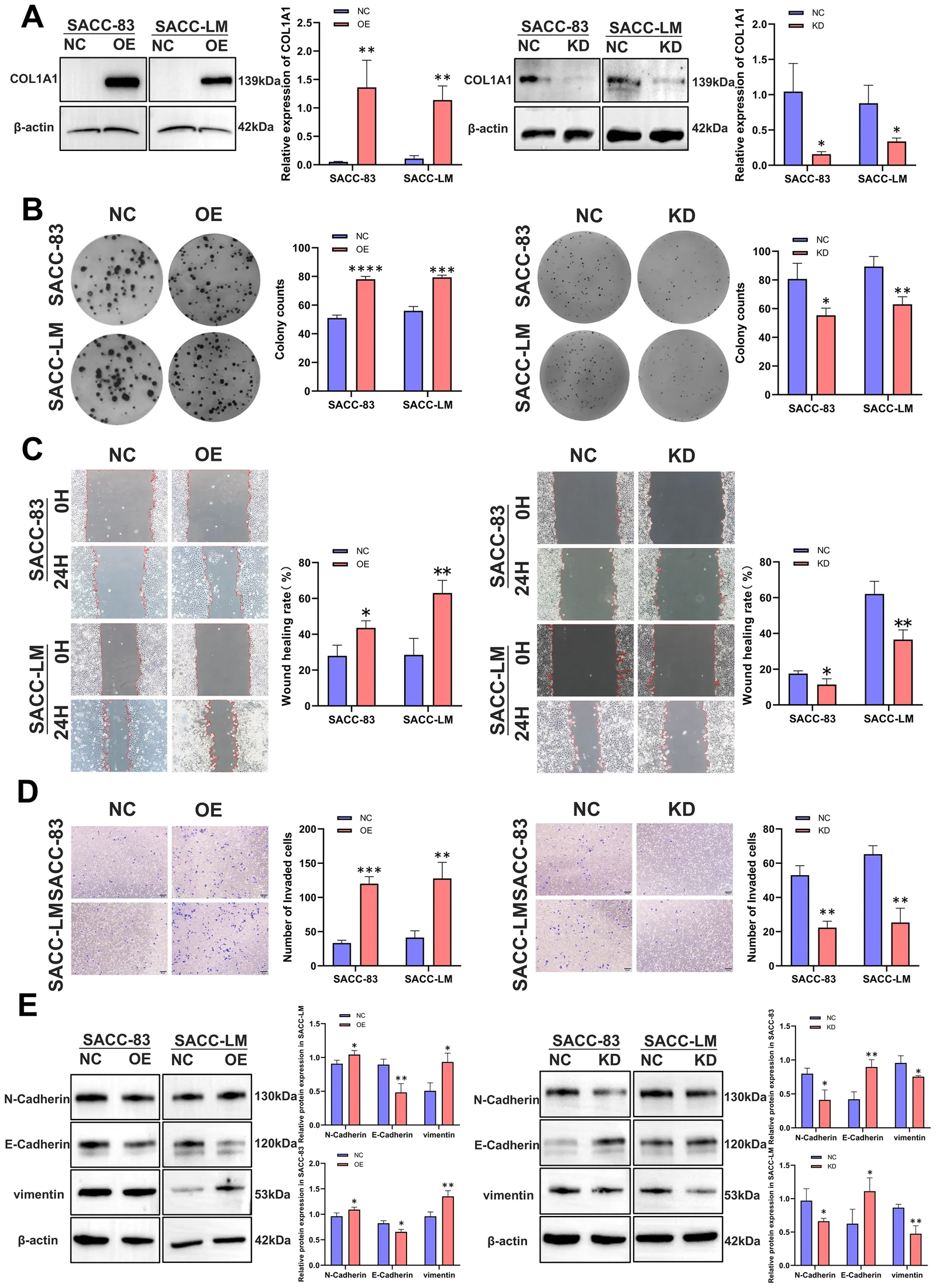
Overexpression of COL1A1 promoted the proliferation, migration, and invasion. **(A)** Western blot analysis results of COL1A1. **(B)** Colony formation assay, **(C)** Wound-healing assay, **(D)** Transwell invasion assay results of SACC-83 and SACC-LM after COL1A1 siRNA or expression plasmid transfection. **(E)** Western blot analysis results of EMT markers. * indicates P < 0.05, ** indicates P < 0.01, *** indicates P < 0.001, and ns indicates no statistically significant difference between groups.

SACC-83 and SACC-LM cells with overexpression or knockdown of COL1A1 were established via transfection with COL1A1-overexpressing lentivirus or *COL1A1* siRNA (**Figure 5A**). Cell experiments revealed that COL1A1 overexpression promoted the proliferation (**Figure 5B**), migration (**Figure 5C**) and invasion abilities (**Figure 5D**) of SACC-83 and SACC-LM cells, whereas COL1A1 knockdown suppressed these malignant cellular functions. As a major component of the extracellular matrix, COL1A1 is closely associated with epithelial-mesenchymal transition (EMT)-dependent tumor metastasis; therefore, we examined the effects of altered COL1A1 expression on EMT markers. Moreover, it was observed that specific knockdown of *COL1A1* in SACC-83 and SACC-LM cells decreased the vimentin and N-cadherin expression while upregulating E-cadherin expression, whereas COL1A1 overexpression yielded the opposite results (**Figure 5E**). These findings suggest that miR-150-5p may regulate EMT by targeting COL1A1, thereby affecting the progression of tumor.

### 3.7 COL1A1 reversed the inhibitory effect of miR-150-5p

**Figure 6:**
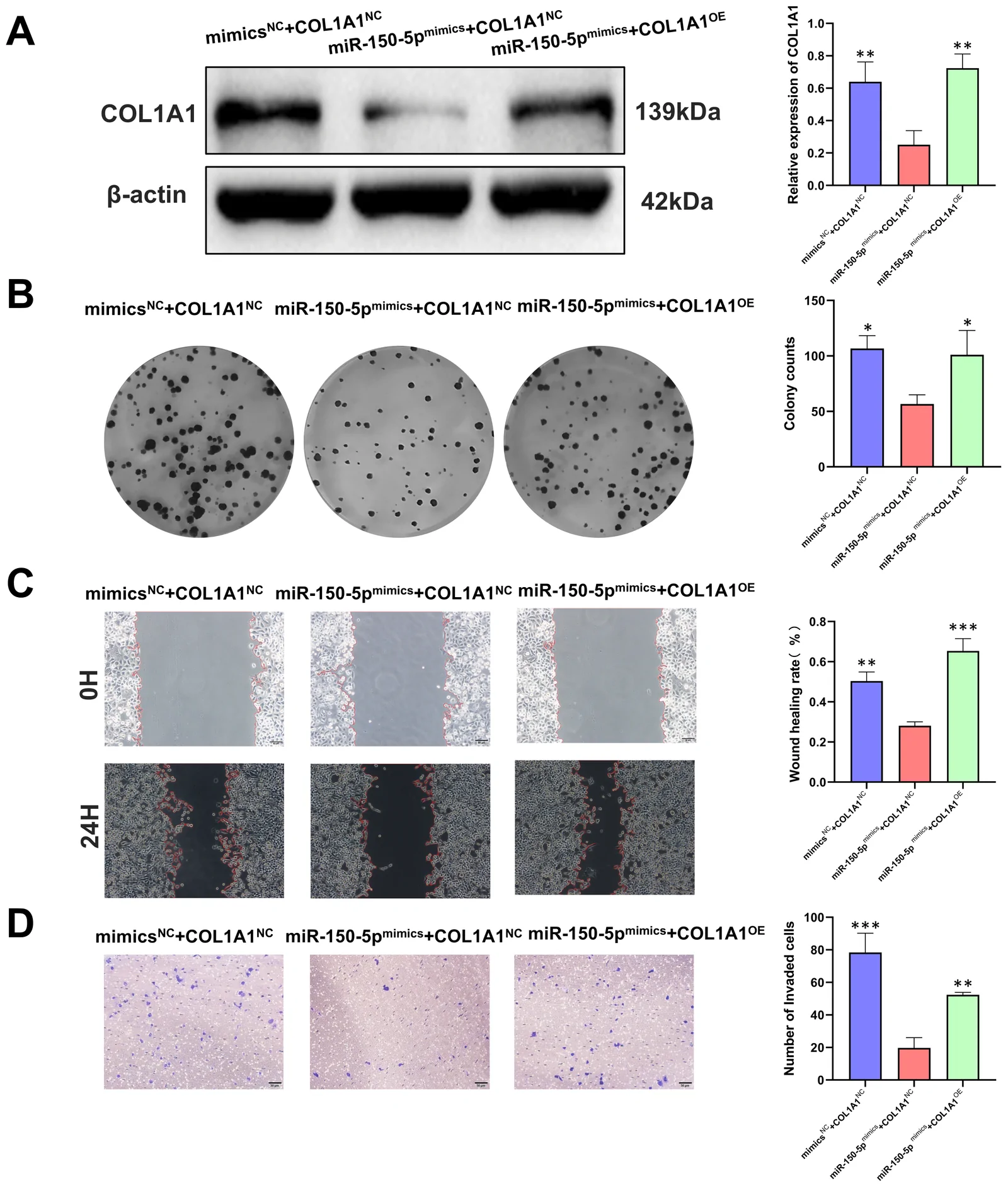
COL1A1 reversed the inhibitory effect of miR-150-5p. **(A)** Western blot analysis results of COL1A1. **(B)** Colony formation assay, **(C)** Wound-healing assay, **(D)** Transwell invasion assay results of SACC-LM after different treatments. * indicates P < 0.05, ** indicates P < 0.01, *** indicates P < 0.001, and ns indicates no statistically significant difference between groups.

To further validate that *COL1A1* is a key downstream target of miR-150-5p, we performed rescue experiments in SACC-LM. When miR-150-5p was overexpressed alone, the protein expression of COL1A1 decreased. However, when both miR-150-5p and *COL1A1* were overexpressed simultaneously, the expression of COL1A1 was relatively restored (**Figure 6A**). Colony formation assays, wound healing assays, and Transwell invasion assays showed that overexpression of COL1A1 could reverse the inhibitory effect of miR-150-5p on cell proliferation (**Figure 6B**), migration (**Figure 6C**), and invasion (**Figure 6D**).

### 3.8 MiR-150-5p suppressed tumor growth *in vivo*

**Figure 7:**
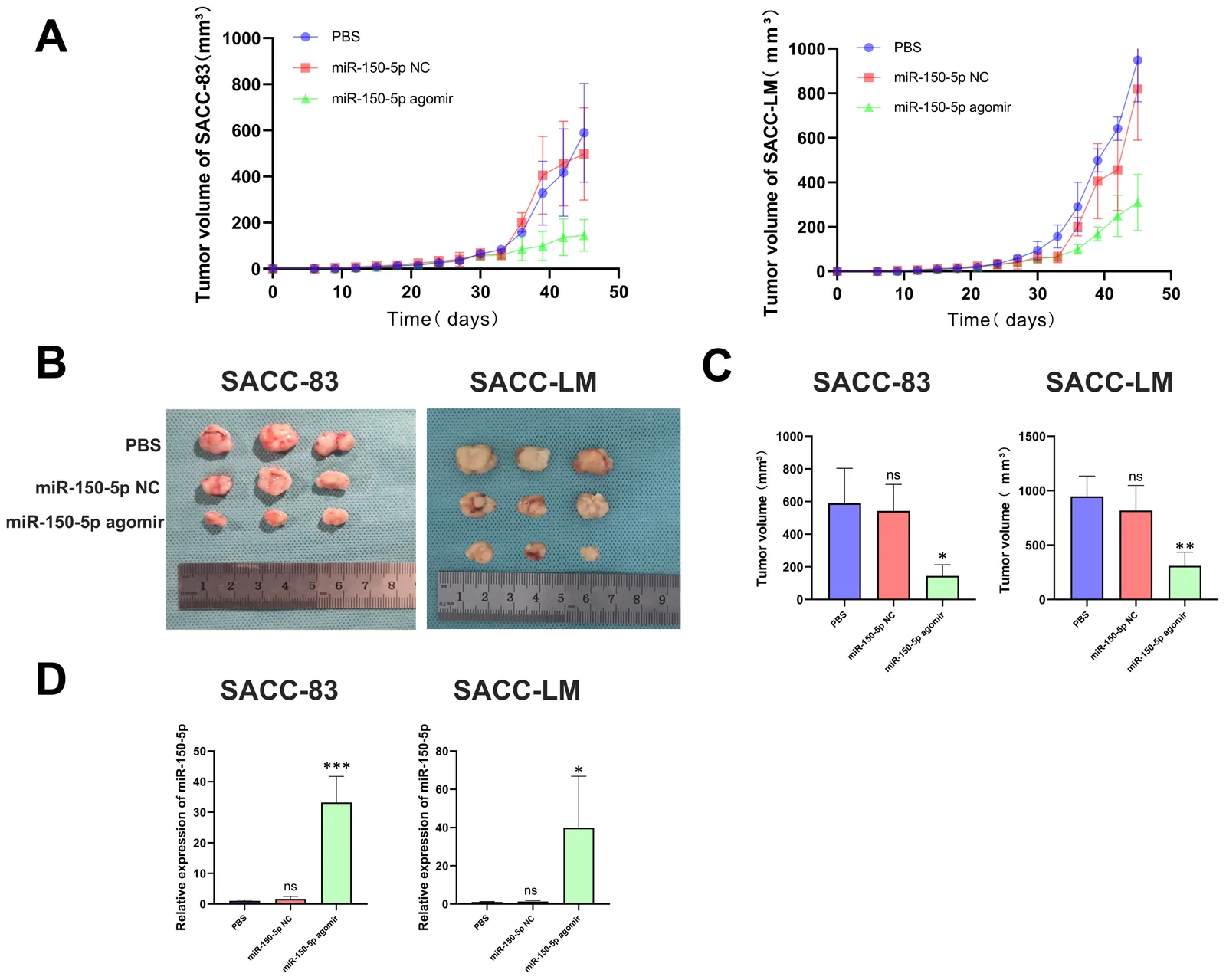
MiR-150-5p suppressed tumor growth *in vivo*. **(A)** Growth curves of SACC-83 and SACC-LM xenograft tumors. **(B)** Solid tumors in SACC-83 and SACC-LM after miR-150-5p overexpression. **(C)** Tumor volume. **(D)** MiR-150-5p expression levels as detected by RT-qPCR.

To investigate the effect of miR-150-5p on tumor proliferation, we constructed the corresponding subcutaneous tumor xenograft models in nude mice by subcutaneously injecting a specific number of SACC-83 and SACC-LM cells, respectively. In both subcutaneous xenograft models, the tumor growth curves (**Figure 7A**) and tumor images showed that the tumor volume in the miR-150-5p agomir group was significantly smaller than those in the other two groups(**Figures 7B, C**), indicating that overexpression of miR-150-5p can inhibit tumor development. RT-qPCR results showed that the expression of miR-150-5p was upregulated in the miR-150-5p agomir group (**Figure 7D**).

## 4 Discussion

LACC is the most common malignant epithelial tumor of the lacrimal gland, characterized by aggressive local invasion, perineural spread, frequent recurrence, and poor long-term survival (7). HGT is a distinct pathological subtype with markedly increased proliferative activity, enhanced metastatic potential and significantly worse clinical outcomes compared with conventional LACC (17, 18). However, the molecular mechanisms underlying HGT remain largely unclear due to the rarity of the disease and limited clinical specimens. In this study, we integrated miRNA microarray profiling, quantitative proteomics, bioinformatic prediction, clinical validation, functional assays, rescue experiments and xenograft models to systematically investigate the role of miR-150-5p in HGT-LACC. Our results showed that miR-150-5p was significantly downregulated in HGT-LACC tissues and correlated with poor prognosis. We further identified *COL1A1* as a direct downstream target of miR-150-5p, and demonstrated that the miR-150-5p/*COL1A1* axis regulates LACC cell proliferation, migration, invasion and EMT. These findings reveal a previously unrecognized regulatory pathway driving HGT-LACC progression.

MicroRNAs are evolutionarily conserved non-coding RNAs that act as pivotal post-transcriptional regulators in tumor initiation, progression, metastasis and therapeutic resistance (19, 20). Among them, miR-150-5p has been widely implicated in cancer biology, predominantly acting as a tumor suppressor in most solid tumors (21–23). Consistent with these reports, our study confirmed the marked downregulation of miR-150-5p in HGT-LACC and its tumor-suppressive function in inhibiting LACC cell proliferation and invasion.

Our findings align with previous studies across multiple epithelial malignancies, while highlighting the tissue-specific nature of miR-150-5p downstream targets. In esophageal squamous cell carcinoma, miR-150 inhibits EMT and tumor progression by targeting *ZEB1 (*21); in hepatocellular carcinoma, it suppresses cell proliferation and metastasis via the *GAB1*/ERK axis (22); in colorectal cancer, it exerts anti-tumor effects through modulation of the Wnt/β-catenin pathway (23). These studies collectively support a conserved tumor-suppressive role for miR-150-5p, but its functional effectors vary substantially across tumor types. Unlike previous reports that identified intracellular signaling molecules or transcription factors as primary targets, our study uncovered COL1A1, a core extracellular matrix (ECM) protein, as the major functional target of miR-150-5p in LACC. This finding expands the biological spectrum of miR-150-5p and suggests that ECM remodeling may represent a dominant mechanism driving HGT-LACC progression.

Although our study primarily focused on identifying the downstream target of miR-150-5p, the mechanisms responsible for its decreased expression during high-grade transformation remain unclear. Previous studies have demonstrated that dysregulation of miRNA expression may result from multiple upstream regulatory mechanisms, including epigenetic modification, transcriptional regulation, and competing endogenous RNA networks (19, 20, 24). In addition, several long non-coding RNAs and circular RNAs have been reported to reduce the biological activity of miR-150-5p by acting as endogenous molecular sponges in different cancer types (25–27). These observations raise the possibility that similar regulatory mechanisms may contribute to the decreased miR-150-5p expression observed in HGT-LACC. However, the upstream regulatory network of miR-150-5p was beyond the scope of the present study and requires further investigation. Future studies integrating transcriptomic profiling, DNA methylation analysis, and non-coding RNA sequencing may help clarify the molecular events responsible for miR-150-5p downregulation during high-grade transformation.

COL1A1 was identified as one of the most significantly upregulated proteins in HGT-LACC via quantitative proteomics, and was further validated as a direct target of miR-150-5p by dual-luciferase reporter assay and gain/loss-of-function experiments. *COL1A1* encodes the α1 chain of type I collagen, a principal structural component of the ECM. Beyond providing mechanical support, dysregulated collagen deposition drives ECM remodeling, alters mechanotransduction and integrin signaling, and actively promotes tumor cell proliferation, migration and invasion (28, 29). Aberrant COL1A1 overexpression has been consistently linked to poor prognosis and increased metastatic potential across multiple solid tumors (30–32). In our cohort, COL1A1 was markedly upregulated in HGT-LACC tissues, inversely correlated with miR-150-5p expression, and predicted unfavorable patient survival, supporting its role as both a downstream effector of miR-150-5p and a potential prognostic biomarker.

Functional experiments further confirmed that COL1A1 promotes malignant phenotypes of LACC cells, and that these effects are closely associated with EMT. EMT is a conserved biological process that enables epithelial cells to acquire mesenchymal characteristics, thereby facilitating tumor invasion and metastatic dissemination (33–35). In our study, COL1A1 overexpression decreased E-cadherin and increased N-cadherin and vimentin, while COL1A1 knockdown produced the opposite effects; consistent changes were observed upon miR-150-5p modulation. These data indicate that the miR-150-5p/*COL1A1* axis regulates LACC progression at least partially through EMT. The precise signaling cascades linking COL1A1 to EMT activation remain to be elucidated in future work.

Rescue experiments and *in vivo* xenograft studies further strengthened the causal relationship between miR-150-5p and COL1A1. Restoration of COL1A1 significantly attenuated the tumor-suppressive effects of miR-150-5p overexpression, confirming *COL1A1* as a major functional downstream target. Notably, the rescue effect was partial rather than complete, consistent with the established concept that a single miRNA typically regulates multiple target genes simultaneously (19, 20, 24), implying that additional effectors of miR-150-5p may also contribute to LACC progression. *In vivo*, intratumoral administration of miR-150-5p agomir significantly inhibited xenograft tumor growth, corroborating the *in vitro* findings and supporting the biological relevance of this regulatory axis.

From a clinical perspective, our findings have several potential implications. Given the rarity of HGT-LACC and the lack of reliable prognostic biomarkers, miR-150-5p and COL1A1 may serve as molecular indicators for risk stratification and individualized patient management. In addition, the *in vivo* anti-tumor effect of miR-150-5p agomir highlights the therapeutic potential of miRNA replacement strategies. Although clinical translation of miRNA-based therapeutics still faces challenges in delivery efficiency and tissue specificity, continuous advances in RNA delivery technologies have greatly promoted the development of this field (36–38), making the miR-150-5p/*COL1A1* axis a promising target for future translational research.

Several limitations of this study should be acknowledged. First, due to the rarity of HGT-LACC, the sample size of our cohort is relatively limited, and multi-center studies with larger populations are needed to validate the prognostic value of miR-150-5p and COL1A1. Second, the upstream mechanisms driving miR-150-5p downregulation during HGT were not experimentally validated in this work. Third, the specific signaling pathways mediating COL1A1-induced EMT remain to be clarified. Finally, the subcutaneous xenograft model cannot fully recapitulate the native tumor microenvironment and metastatic behavior of primary LACC; more physiologically relevant models such as orthotopic xenografts or patient-derived xenografts will provide deeper insights in future studies.

## 5 Conclusions

In conclusion, our study demonstrates that miR-150-5p functions as a tumor suppressor in HGT-LACC by directly targeting *COL1A1* and inhibiting EMT-associated malignant progression. The miR-150-5p/*COL1A1* axis represents a previously unrecognized molecular pathway driving high-grade transformation, providing novel insights into LACC pathogenesis and potential biomarkers and therapeutic targets for this aggressive malignancy.

## Data Availability

The data presented in the study are deposited in the Figshare repository, DOI: 10.6084/m9.figshare.33295953.
